# Gelatin-Based Hydrogels Promote Chondrogenic Differentiation of Human Adipose Tissue-Derived Mesenchymal Stem Cells *In Vitro*

**DOI:** 10.3390/ma7021342

**Published:** 2014-02-19

**Authors:** Achim Salamon, Sandra van Vlierberghe, Ine van Nieuwenhove, Frank Baudisch, Geert-Jan Graulus, Verena Benecke, Kristin Alberti, Hans-Georg Neumann, Joachim Rychly, José C. Martins, Peter Dubruel, Kirsten Peters

**Affiliations:** 1 Department of Cell Biology, Rostock University Medical Center, Schillingallee 69, Rostock D-18057, Germany; E-Mails: frank.baudisch@gmail.com (F.B.); verena.benecke@web.de (V.B.); joachim.rychly@med.uni-rostock.de (J.R.); kirsten.peters@med.uni-rostock.de (K.P.); 2 Polymer Chemistry and Biomaterials Group, Gent University, Krijgslaan 281, Building S4, Gent BE-9000, Belgium; E-Mails: ine.vannieuwenhove@ugent.be (I.N.); geertjan.graulus@ugent.be (G.-J.G.); peter.dubruel@ugent.be (P.D.); 3 DOT GmbH, Charles-Darwin-Ring 1a, Rostock D-18059, Germany; E-Mail: neumann@dot-coating.de; 4 NMR and Structure Analysis Research Group, Gent University, Krijgslaan 281, Building S4, Gent BE-9000, Belgium; E-Mail: jose.martins@ugent.be

**Keywords:** adipose tissue-derived mesenchymal stem cells (adMSC), gelatin-based hydrogels, chondrogenic differentiation, tissue engineering

## Abstract

Due to the weak regeneration potential of cartilage, there is a high clinical incidence of articular joint disease, leading to a strong demand for cartilaginous tissue surrogates. The aim of this study was to evaluate a gelatin-based hydrogel for its suitability to support chondrogenic differentiation of human mesenchymal stem cells. Gelatin-based hydrogels are biodegradable, show high biocompatibility, and offer possibilities to introduce functional groups and/or ligands. In order to prove their chondrogenesis-supporting potential, a hydrogel film was developed and compared with standard cell culture polystyrene regarding the differentiation behavior of human mesenchymal stem cells. Cellular basis for this study were human adipose tissue-derived mesenchymal stem cells, which exhibit differentiation potential along the adipogenic, osteogenic and chondrogenic lineage. The results obtained show a promotive effect of gelatin-based hydrogels on chondrogenic differentiation of mesenchymal stem cells *in vitro* and therefore encourage subsequent *in vivo* studies.

## 1. Introduction

Articular cartilage has only weak regeneration potential [1]. Following traumatic or degenerative cartilage injury, this leads to a strong clinical demand for cartilaginous tissue surrogates. Since current strategies of autologous chondrocyte implantation suffer from chondrocyte de-differentiation following expansion *in vitro* [2], stem cells in conjunction with biomaterial scaffolds are quested to both accelerate and improve cartilage tissue repair [3]. The aim of the present study was to evaluate a new approach for cartilage tissue regeneration based on the utilization of mesenchymal stem cells (MSC) in contact to biodegradable gelatin-based hydrogels as supporting matrix.

The main constituent of the hydrogels developed is gelatin, a non-toxic, biodegradable and water-soluble protein derived from collagen, with the latter being a major component of mesenchymal tissue extracellular matrix (ECM). Gelatin retains informational signals including an arginine-glycine-aspartic acid (RGD) sequence, which promotes cell adhesion, proliferation and stem cell differentiation [4]. According to the production process, two types of gelatin with different isoelectric points can be distinguished [5]. Via acidic hydrolysis of collagen, gelatin type A with an isoelectric point of 6 to 8 is obtained. Conversely, an alkaline collagen treatment results in the development of gelatin type B with an isoelectric point of 4.7 to 5.3 [6]. In the present work, gelatin type B was selected as starting material, exhibiting superior biocompatibility compared to gelatin type A due to the different treatment conditions applied [7]. A characteristic property of gelatin is that it exhibits Upper Critical Solution Temperature behavior (UCST). Above a specific temperature threshold of 40 °C, gelatin can be dissolved in water by the formation of flexible, random single coils. Upon cooling, hydrogen bonding and Van der Waals interactions occur, resulting in the formation of triple helices. These collagen-like triple helices act as junction zones and thus trigger the sol-gel transition. However, in the scope of tissue engineering applications, chemical crosslinks still need to be incorporated to ensure stability around body temperature, as the physical crosslinks melt at these temperatures [8]. To date, several crosslinking strategies have already been elaborated. For example, Van Vlierberghe *et al*.[8] applied thiol functionalities that can be oxidized to disulfides in the presence of a suitable oxidant such as hydrogen peroxide. However, as unprotected thiols are sensitive to crosslinking in the presence of oxygen, hydrogel processing is not very straightforward in an air environment. Alternatively, a conventional carbodiimide crosslinking strategy is often applied, combining 1-ethyl-3-(3-dimethylaminopropyl) carbodiimide (EDC) with N-hydroxysuccinimide (NHS) in order to covalently link the amines present in gelatin with the activated carboxylic acids [9–11]. In the latter case however, the processed hydrogel structure typically is incubated in a solution containing EDC and NHS. As a result, the crosslinking kinetics are of utmost importance, as gelatin also swells in an aqueous environment. Furthermore, genipin, a natural crosslinker extracted from gardenia fruit, has already been reported on as well with respect to gelatin crosslinking, as it combines two primary amines functions [12]. Unfortunately, this crosslinker typically requires longer crosslinking times. In the present work, gelatin was functionalized with methacrylamide side groups, enabling the chemical crosslinking upon applying UV-irradiation in the presence of a UV-active photo-initiator. The above-mentioned properties including its unique gel-forming ability render gelatin an interesting biopolymer towards tissue engineering applications.

MSC are progenitor cells resident in specialized niches within mesenchymal tissues *in vivo* and govern tissue regeneration by their intrinsic capacity for self-renewal and multipotent mesenchymal differentiation [13,14]. MSC were discovered within the bone marrow stroma [15] as adventitial cells on the outer surface of sinusoidal blood vessels [16]. Later on, the perivasculature was recognized to be the characteristic niche of all MSC independent of their tissue origin [17]. In this study, we used adipose tissue-derived MSC (adMSC). adMSC make up 7% of the cells found in liposuction-derived and collagenase-digested adipose tissue [18] and being present at a concentration of roughly 50,000 cells per ml tissue [19], which is 100-fold higher than that of bone marrow-derived MSC (bmMSC) [20]. Human adMSC were shown to directly differentiate into osteoblasts and endothelial cells in a non-union fracture model in SCID mice [20] and in immunodeficient rats [21]. In that manner, adMSC were demonstrated to reconstitute bone physiology in mice [20], rats [21,22], pigs [23] and dogs [24]. In human case studies, human adMSC were already successfully used to treat critical size calvarial [25] and maxilla defects [26] that, due to the limitation in the amount of auto-graftable bone tissue, could otherwise not have been healed. Finally, adMSC were demonstrated to engraft at long-term at the transplantation site and to migrate to and survive within the perivascular MSC niche [20–22,27]. Therefore, adMSC fulfill all MSC characteristics defined by the International Society for Cellular Therapy, the International Federation of Adipose Therapeutics and Sciences, and others [28–30] and thus are a crucial cell type for regenerative medicine.

## 2. Experimental Section

### 2.1. Preparation of Chemically Crosslinked Gelatin Hydrogels

Gelatin type B, isolated from bovine skin, was kindly supplied by Rousselot (Ghent, Belgium). Gelatin samples with an approximate isoelectric point of 5 and a Bloom strength of 257 were used. Methacrylic anhydride (MAA) was purchased from Aldrich (Bornem, Belgium) and was applied as received. Dialysis membranes Spectra/Por 4 (MWCO 12,000 – 14,000 Da) were obtained from Polylab (Antwerp, Belgium). 1-[4-(2-Hydroxyethoxy)-phenyl]-2-hydroxy-2-methyl-1-propane-1-one (Irgacure 2959) was a kind gift from Ciba Specialty Chemicals N.V. (Groot-Bijgaarden, Belgium). A LWUV-lamp model VL-400L (Vilber Lourmat, Marne La Vallee, France) with an intensity of 10 mW/cm^2^ and a wavelength range of 250 – 450 nm was applied in order to cure the hydrogels.

Gelatin was dissolved in PBS buffer (pH 7.3) at 40 °C. While vigorously stirring, 1 equivalent methacrylic anhydride relative to the amine functionalities present in gelatin B was added. After 1 h reaction time, the methacrylamide-modified gelatin (gel-MOD, see reaction scheme below) was purified via dialysis (MWCO: 12,000 – 14,000 Da), followed by freeze-drying. Interestingly, the amount of crosslinkable side groups can be adjusted by varying the amount of methacrylic anhydride added. In the present work, a modification degree of 60% was applied, which was obtained by the addition of one equivalent methacrylic anhydride.

### 2.2. Characterization of Gelatin-Based Hydrogels

#### 2.2.1. HR-MAS Spectroscopy

High Resolution Magic Angle Spinning NMR spectroscopy (HR-MAS NMR) of the developed hydrogel films was performed on a Bruker Avance II 700 spectrometer (700.13 MHz) using a HR-MAS probe equipped with a ^1^H, ^13^C, ^119^Sn and gradient coil. The spinning rate was adjusted to 6 kHz. HR-MAS samples were obtained by placing a small amount of the freeze-dried hydrogels inside a 4 mm zirconium oxide MAS rotor (50 μL). A few microliter of deuterium oxide (D_2_O) were added to the rotor, allowing the samples to swell. The samples were homogenized by manual stirring prior to analysis. A teflon-coated cap was used to close the rotor.

#### 2.2.2. X-ray Photoelectron Spectroscopy

X-ray photoelectron spectroscopy (XPS) was applied as a non-destructive, quantitative spectroscopic technique to determine the chemical composition of native and functionalized gelatin. A FISONS S-PROBE with a fine focus Al-Kα source (1486 eV) and a quartz monochromator developed by Fisons Instruments Surface Science (now Sanofi, Paris, France) was used for the measurement performed using a dedicated XPS instrument able to give the ultimate in performance, while providing a high sample throughput. All measurements were done in triplicate in a vacuum of at least 10^−9^ Pa. Wide and narrow-scan spectra were acquired at pass energies of 158 and 56 eV, respectively. The binding energy was calibrated by the C1s peak at 284.6 eV, and the utilized spot size was 250 μm on 1 mm. Data analysis was performed using S-PROBE software.

### 2.3. adMSC Isolation and Culture

If not stated otherwise, all plasticware was from Greiner Bio-One (Frickenhausen, Germany), whereas all reagents were from Sigma-Aldrich (Taufkirchen, Germany).

adMSC were isolated from adipose tissue of healthy patients having undergone tumescence-based liposuction. As described previously [31], adipose tissue was digested using Collagenase NB4 (SERVA, Heidelberg, Germany), a mixture of a neutral protease and the collagenases I and II (6 mg/mL in phosphate-buffered saline (PBS); PAA Laboratories, Cölbe, Germany). The cells thus liberated from the digested tissue were incubated over night under standard cell culture conditions (5% CO_2_ and 37 °C in a humidified atmosphere). CD34^+^ adventitial cells were isolated from total plastic-adherent cells using an antibody-magnetizable bead-based system (Life Technologies, Darmstadt, Germany) [32]. After three passages, adMSC were seeded into experimentation at 20,000 cells per cm^2^. Expression of MSC markers CD29, CD44, CD105, and CD166, and absence of CD14^+^/CD68^+^ monocytes/macrophages and CD31^+^ endothelial cells as well as absence of *Mycoplasma* species was confirmed.

The adMSC cultivation media used were *u*n*s*timulated control medium (US) and *c*hondrogenic differentiation *s*timulating medium (CS). US was Dulbecco’s Modified Eagle Medium (DMEM), high glucose, GlutaMAX-I, supplemented with 1% penicillin/streptomycin (100 U/mL and 100 μg/mL, respectively; both Life Technologies), and 10% fetal calf serum (FCS; PAN Biotech, Aidenbach, Germany). CS medium was serum-free US medium supplemented with 100 nM dexamethasone, 50 μg/mL ascorbic acid, 50 ng/mL insulin-like growth factor 1 (R&D Systems, Wiesbaden-Nordenstadt, Germany), 50 ng/mL TGF beta 1 (PeproTech, Hamburg, Germany) and 5 μg/mL insulin, 5 μg/mL transferrin and 5 ng/mL selenious acid (the latter three: BD Biosciences, Heidelberg, Germany). At confluence, culture with distinct differentiation-inducing media began, termed day zero, and took up to 28 days. Renewal of medium was every second to third day.

### 2.4. Ethical Statement

adMSC were isolated from patient tissue after written informed consent had been obtained that patients are willing to participate in this study. The study was approved by the ethics committee of Rostock University Medical Center (http://www.ethik.med.uni-rostock.de/) under the registration numbers A17/2007 and A2013-0112, and it complies with the ethical standards defined by the World Medical Association Declaration of Helsinki.

### 2.5. Analysis of adMSC Chondrogenic Differentiation

#### 2.5.1. Live-Dead Staining of adMSC on Hydrogels

adMSC were seeded onto TCPS and hydrogels and cultured for four weeks in US and CS medium. For live-dead staining, cells were incubated for 10 min under standard culture conditions with US medium supplemented with 3 μM Hoechst 33342, 500 nM propidium iodide and 1 μM calcein acetoxymethyl ester (calcein AM, from Life Technologies, Carlsbad, CA, USA). After medium exchange for US medium, cells were subjected to fluorescence microscopy (Carl Zeiss MicroImaging, Göttingen, Germany) using the blue, green and red emission filters. Emission maxima of the dyes are 460 nm (DAPI), 516 nm (calcein), and 617 nm (propidium iodide). Excitation maxima are 360 nm (DAPI), 496 nm (calcein) and 535 nm (propidium iodide).

#### 2.5.2. Quantification of Chondrogenic Marker Gene Expression

Gene expression analysis was done after 14 and 28 days of adMSC culture on TCPS and hydrogels in US and CS medium, employing real-time reverse transcriptase polymerase chain reaction (real-time RT PCR). Briefly, cells were homogenized by centrifugation on QIAshredder columns (Qiagen, Hilden, Germany), and RNA was isolated from the lysate using the innuPREP RNA Mini Kit (Analytik Jena, Jena, Germany) following the manufacturer’s instructions. Subsequently, RNA was transcribed into cDNA using the QuantiTect Reverse Transcription Kit (Qiagen). Real-time PCR of transcribed samples was then performed on a 7500 Real Time PCR system using the Power SYBR Green PCR Master Mix (both Life Technologies). Expression of chondrogenic marker genes was determined using the primers listed in Table 1:

Both cDNA-specific primers were designed using the Primer3 engine [33]. Gene expression levels were determined by the ∆∆*C_t_* method, normalizing to the expression of actin, beta (ACTB).

#### 2.5.3. Staining of Cartilaginous Matrix Proteins

Acidic polysaccharides present in cartilage, which mostly are glycosaminoglycans, were stained after 28 days of adMSC culture using Alcian Blue, resulting in a greenish-blue staining of the extracellular matrix. Briefly, cells were fixed with 4% paraformaldehyde for 1 h at room temperature, washed 3*×* in 2% acetic acid and incubated over night at room temperature in Alcian Blue staining solution (1 mg/mL Alcian Blue 8 (pH = 2) mixed isovolumetrically with 80 mg/mL MgCl_2_, both in 0.9% NaCl). After washing 3*×* with 2% acetic acid and 3*×* with double distilled water (ddH_2_O), cells were subjected to bright-field phase contrast microscopy (Carl Zeiss MicroImaging).

### 2.6. Data Illustration and Statistics

Data are illustrated as box plots created using R [34]. Since the data obtained were in most cases not normally distributed, testing for significance in the difference between two datasets was done using the non-parametric Mann-Whitney *U* test. The level of significance was set to a *p*-value of lower or equal 0.05 (*p ≤* 0.05) and determined using R.

## 3. Results and Discussion

### 3.1. Hydrogel Physico-Chemical Characterization

Crosslinking of a 10% (w/v) gelatin-methacrylamide (gel-MOD) solution was done via photo-initiation upon applying UV-irradiation in the presence of 2 mol% Irgacure 2959 (with respect to the methacrylamide side chains present). The polymer solutions were injected between two parallel glass plates separated by a 1 mm thick silicone spacer. The materials were allowed to form gels after applying UV-A irradiation.

High Resolution Magic Angle Spinning Nuclear Magnetic Resonance (HR-MAS NMR) spectroscopy was applied as a straightforward technique to evaluate the crosslinking efficiency of the UV-cured hydrogels. Conventional ^1^H-NMR spectroscopy does not enable the characterization of water-insoluble polymer networks due to considerable line broadening resulting from the presence of dipolar couplings and magnetic susceptibility [35,36]. However, this broadening can be circumvented by rapidly rotating (in the order of kilohertz) the sample at a magic angle *θ* of 54.7° with respect to the static magnetic field [36]. In this way, the contribution of the line broadening effects defined by a (3 cos^2^ *θ* *−* 1)*/*2 orientation dependence is removed from the spectrum. Further decrease in line width is realized by swelling the hydrogel samples in a suitable deuterated solvent resulting in sufficient, solution-like, rotational mobility of the polymer [37]. As a consequence, highly crosslinked hydrogel materials exhibiting a reduced chain mobility will show broader peaks than less crosslinked materials [38].

The crosslinking efficiency can be determined by comparing the integration of the signal corresponding to the double bonds with the integration of a signal that remains chemically inert during crosslinking. Upon crosslinking, the introduced double bonds are consumed, enabling determination of the crosslinking efficiency (*CE*). The *CE* is calculated by comparing the intensity of the signals characterizing the protons of the double bonds, before and after crosslinking. Since different samples have to be compared, normalization is required using the inert signal (*i.e.*, at 1.1 ppm). The crosslinking efficiency for the cured gelatin hydrogels is given by the following formula [37], where the superscript i stands for initial, non-crosslinked and c for crosslinked:

$$CE\;(\%)=\left(\frac{\frac{I_{5.75\;\;\text{or}\;\;5.1\;\text{ppm}}^{i}}{I_{1.1\;\text{ppm}}^{i}}-\frac{I_{5.75\;\;\text{or}\;\;5.51\;\text{ppm}}^{c}}{I_{1.1\;\text{ppm}}^{c}}}{\frac{I_{5.75\;\;\text{or}\;\;5.51\;\text{ppm}}^{i}}{I_{1.1\;\text{ppm}}^{i}}}\right).100$$

**Figure 1.**
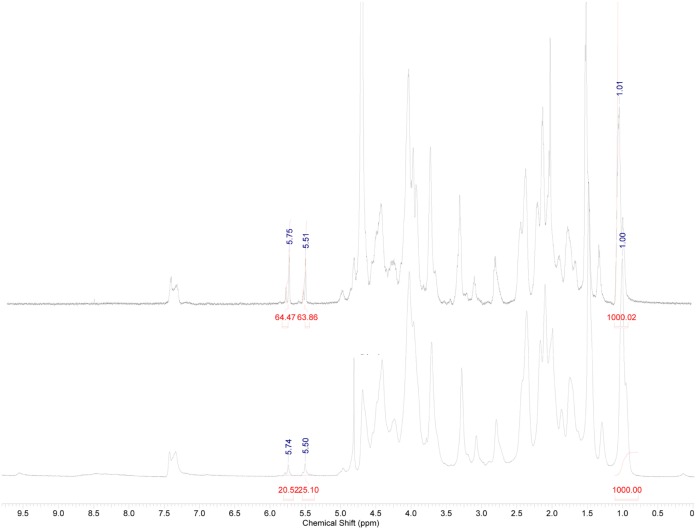
1H-Nuclear Magnetic Resonance (NMR) spectrum of non-crosslinked gelatin-methacrylamide (**top**) and High Resolution Magic Angle Spinning (HR-MAS) NMR spectrum of crosslinked gelatin-methacrylamide (**bottom**).

HR-MAS spectroscopy indicated an efficient UV-crosslinking for the gelatin-based hydrogels as a *CE* of 69% was obtained (see Figure 1).

In a subsequent part of the present work, the effect of the functionalization on the chemical composition of gelatin was determined via XPS measurements. Table 2 shows an overview of the atomic composition of gelatin and gel-MOD. The results indicate that Gel-MOD exhibits lower values compared to gelatin for the N/C ratio (*i.e.*, 0.12 *versus* 0.19) and the N/O ratio (*i.e.*, 0.56 *versus* 0.72), as anticipated based on the fact that methacrylamide moieties are introduced on the primary amines of (hydroxy-)lysine upon modification. For each primary amine in the gelatin backbone that is converted into methacrylamide, three additional carbons and one additional oxygen are introduced (see reaction scheme on page 1345). The results therefore indicate that the gelatin modification was successful.

### 3.2. adMSC Chondrogenic Differentiation on Hydrogels

#### 3.2.1. Live-Dead Staining of adMSC on Hydrogels

**Figure 2.**
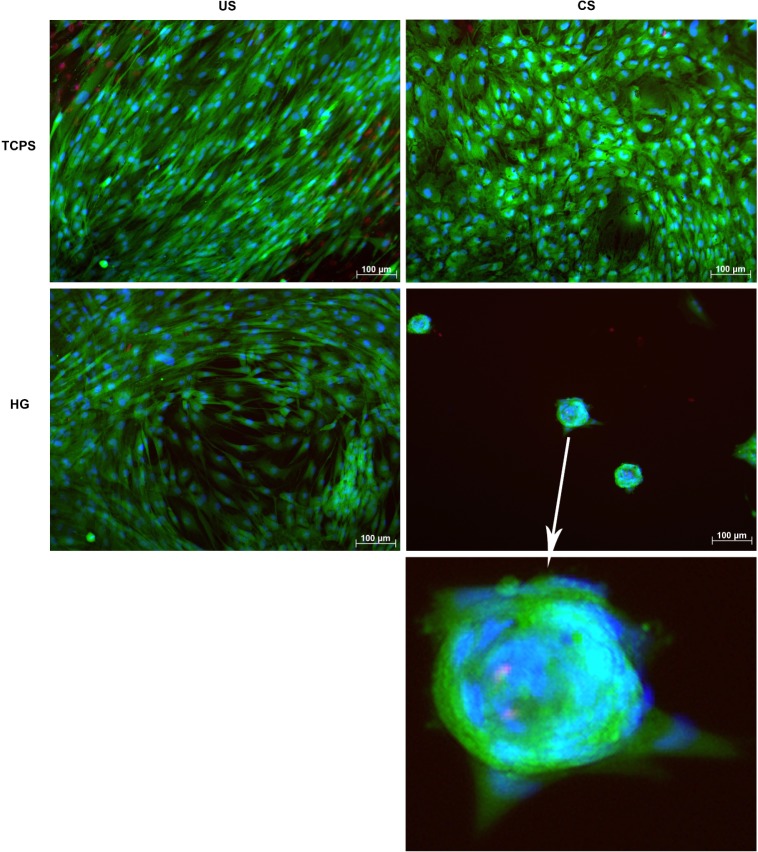
Phenotype of adMSC on hydrogels after 28 days of chondrogenic stimulation. adMSC were seeded onto hydrogels (HG) at 20,000 cells/cm^2^, tissue culture polystyrene (TCPS) serving as a control. After cell attachment, medium was exchanged and cells were grown to a confluent layer. At that point, chondrogenic stimulation (CS) was started and done for 28 days, exchanging cell culture medium every second to third day and using unsupplemented medium as unstimulated control (US). Live cell staining was with calcein AM (green fluorescence), dead cell staining was with propidium iodide (red fluorescence), and total cell nuclei staining was with Hoechst33342 (blue fluorescence).

For testing of cytocompatibility and phenotypical changes upon differentiation-inducing conditions, human adMSC were live dead-stained following seeding onto hydrogels pre-incubated for 2 h with cell culture medium. Tissue culture polystyrene (TCPS) was used as a control adhesion surface. After 28 days of chondrogenic stimulation (CS) on TCPS, adMSC showed a more condensed and cobblestone-like phenotype with distinct areas of cell aggregation, which was not the case for the unstimulated (US) control adMSC being evenly spread and showing a spindle-like shape typical for cells of the fibroblastoid lineage (Figure 2). On the hydrogels without chondrogenic stimulation, adMSC tended to aggregate, but still showed a spindle-like phenotype, while chondrogenic stimulation of adMSC on the hydrogels resulted in strong aggregation into spheroid-like structures of approximately 70 μm diameter.

The cellular condensation step observed on the hydrogels after chondrogenic stimulation resembles the process of mesenchymal condensation, *i.e.*, the aggregation of cells into three-dimensional structures that subsequently build up the distinct layers of cell-extracellular matrix composites found in cartilaginous tissue [39,40]. Mesenchymal condensation is a prerequisite for chondrogenic differentiation to occur [41–43] and depends on matrix elasticity. Human bmMSC on flexible silk fibroin matrices with a lateral elasticity modulus of 4.8 GPa were found to maintain a rounded cell phenotype, to promote cell aggregation in chondrogenic medium and to significantly increase cartilaginous type II collagen and glycosaminoglycan accumulation as well as *SOX9* expression, while stiffer matrices of 7.8 GPa composed of identical material supported a flattened cell phenotype without cellular aggregation and increased cartilage marker expression [44]. Thus, the gelatin-based hydrogels used here promote adMSC condensation, but require additional chondrogenic stimulation to fully induce this differentiation pathway.

#### 3.2.2. Staining of Cartilaginous Matrix Proteins

**Figure 3.**
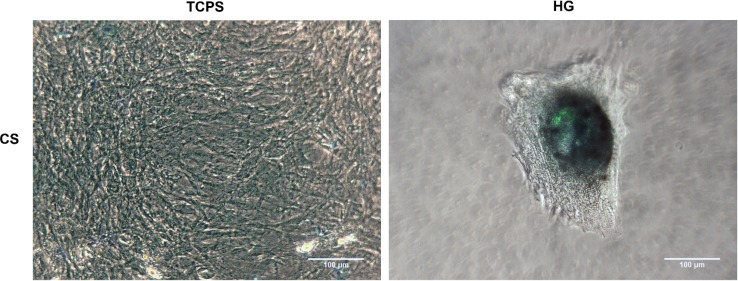
Acidic polysaccharide staining of chondrogenically stimulated adMSC on hydrogels after 28 days of stimulation. Chondrogenically stimulated (CS) adMSC on tissue culture polystyrene (TCPS) or on hydrogels (HG) were stained for the presence of glycosaminoglycans using Alcian Blue and visualized by bright-field phase contrast microscopy. The scale bar is 100 μm.

Having noticed that chondrogenically stimulated adMSC aggregated into spheroid-like structures on the hydrogels, we looked for the presence of acidic polysaccharides characteristic for cartilaginous tissue, since cartilage extracellular matrix characteristically consists of proteins glycosylated by glycosaminoglycans, so-called proteoglycans as e.g., aggrecan (ACAN) [2,42]. Staining of acidic glycosaminoglycans on the surface and within the hydrogels was done using Alcian Blue [45,46]. Following Alcian Blue staining, we found a strong accumulation of glycosaminoglycans inside the spheroids formed by chondrogenically stimulated adMSC on the hydrogels, while the even cell layer formed by chondrogenically stimulated adMSC on TCPS showed negligible staining (Figure 3).

Thus, hydrogels, in combination with chondrogenic stimulants, have a strong inductive effect on the deposition of cartilaginous matrix components by adMSC.

#### 3.2.3. Expression of Chondrogenic Marker Genes

**Figure 4.**
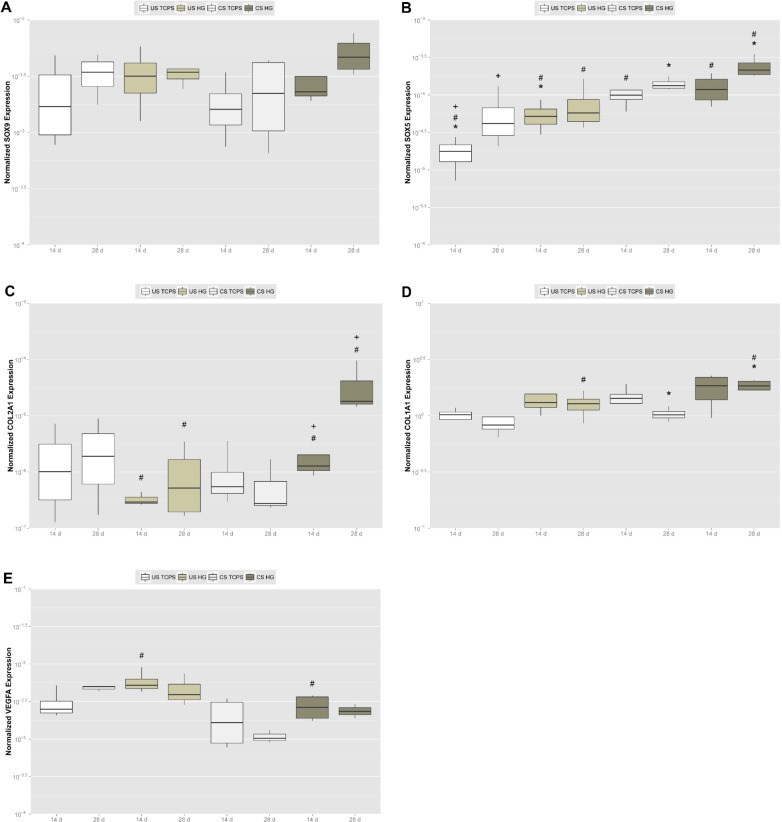
Analysis of chondrogenic marker gene expression by adMSC on hydrogels after 14 and 28 days of chondrogenic stimulation. Gene expression was analyzed by real-time RT PCR, normalizing to the expression of the beta actin gene *ACTB*. We analyzed expression of transcription factors sex determining region Y box 9 (*SOX9*) and 5 (*SOX5*) (subfigures (**A**) and (**B**), respectively), collagen type II, alpha 1 (*COL2A1*) and type I, alpha 1 (*COL1A1*) (subfigures (**C**) and (**D**), respectively), and vascular endothelial growth factor A (*VEGFA*) (subfigure (**E**)). Mann-Whitney *U* test, *p ≤* 0.05. * TCPS significantly different to HG, # US significantly different to CS, + 14 days significantly different to 28 days.

To more closely and quantitatively investigate onset and progression of chondrogenic differentiation of adMSC on the hydrogels, we analyzed expression of genes involved in that process using real-time RT PCR. Expression of *SOX9*, a gene encoding a member of the SOX (SRY-related HMG-box) family, was upregulated on hydrogels and following chondrogenic stimulation (Figure 4A). The strongest *SOX9* expression was found for chondrogenically stimulated adMSC on the hydrogels after 28 days. Expression of the transcription factor *SOX5* gene, another member of the HMG-box class DNA-binding proteins, was significantly and time-dependently upregulated on the hydrogels as compared to TCPS (Figure 4B). Upon chondrogenic stimulation of adMSC, expression of this gene significantly increased, an effect that was the strongest on the hydrogels.

The trio of SOX transcription factors 5, 6 and 9 plays a crucial role in chondrogenesis, SOX9 being the mayor player and the single factor containing a transactivation domain, while complexation with SOX5 and 6, which contain only DNA-binding domains, has a promotive effect on cooperative binding of the homodimerized SOX trio to enhancers of chondrocyte-specific genes [47]. Moreover, SOX9 upregulates expression of *SOX5* and *SOX6* [43] and is required for chondrogenic lineage commitment by MSC condensation, its expression shutting down at the prehypertrophic stage, while the auxiliary *SOX5* is expressed in a constitutive manner, being activated in prechondrocytes and highly expressed until the chondroblast stage [41–43]. Chondrogenic stimulation using transforming growth factors beta 1 (TGFβ1) or 3 (TGFβ3) was shown to upregulate SOX9, while osteogenic stimulation via the Wingless Int-1 pathway (WNT) downregulates this factor [43]. Simultaneously, SOX9 was reported to inhibit the osteoblastogenesis key transcription factor runt-related transcription factor 2 (RUNX2) [40]. Therefore, our chondrogenic transcription factor gene expression data underline a promotive impact of the hydrogels on chondrogenic differentiation of adMSC *in vitro*.

This supportive impact of the hydrogels is sustained by the data obtained for collagen gene expression. Cartilage consists primarily of type II collagen that entraps hydrated proteoglycans and glycoproteins to build a gel-like extracellular matrix, and the SOX trio of transcription factors binds to a type II collagen enhancer to directly drive expression of *COL2A1*, the gene encoding the alpha-1 chain of type II collagen [41–43]. In our experiments, *COL2A1* expression was significantly and time-dependently upregulated for chondrogenically stimulated adMSC on the hydrogels, but downregulated on TCPS (Figure 4C). Expression of *COL1A1*, the gene encoding the pro-alpha-1 chains of the fibril-forming collagen type I found most connective tissues, was significantly upregulated upon chondrogenic stimulation, an effect that was significantly stronger on the hydrogels (Figure 4D). Since most cartilage *in vivo* is articular cartilage located in between joint space and bone and type I collagen is the mayor collagen type found in bone [39,48], our gene expression data for type I and II collagen clearly point out a promotive effect of the hydrogels on chondrogenic differentiation of adMSC *in vitro*.

Finally, we found expression of the *VEGFA* gene to be strongly upregulated for adMSC on the hydrogels, but to be significantly downregulated when cells were stimulated chondrogenically (see Figure 4E). Vascular endothelial growth factor A (VEGFA) is a prominent player in vasculogenesis whose expression is induced by SOX9 [49] and COL2A1 [50], but most interestingly by the hypoxia-induced transcription factor hypoxia inducible factor 1 alpha (HIF1A), as shown in rat PC12 cells [51], in human pulmonary artery endothelial cells [52] and in human Hep3B cells [53]. Within the avascular and alymphatic cartilaginous tissue [1], HIF1A was shown to be required for endochondral ossification to proceed [39], being a critical survival factor for hypoxic chondrocytes and increasing cartilaginous extracellular matrix accumulation and early chondrogenesis [50]. Thus, the strong upregulation of *VEGFA* expression for unstimulated adMSC on the hydrogels may reflect the onset of gene expression to counteract hypoxic conditions occurring in the center of the spheroids formed, while additionally stimulating chondrogenically abolishes pro-angiogenic gene expression despite central hypoxia and therefore resembles chondrogenesis *in vivo*.

## 4. Conclusions

Hence, our gene and protein expression data argue in favor of a pro-chondrogenic impact of gelatin-based hydrogels on adMSC *in vitro*. Data on chondrogenic differentiation of MSC on gelatin-based hydrogels are rare in the literature [8]. Commonly used naturally-derived hydrogels in cartilage studies are based on collagen and alginate [48] or on hydroxyapatite [1]. In these studies, stronger cartilaginous extracellular matrix synthesis and a more physiologic compressibility were observed. However, proteoglycan content was only 10% of that present in the *nucleus pulposus* of intervertebral discs *in vivo*, which leaves a great need for improvement. Initial studies employing rabbit bmMSC on an osteochondral composite material consisting of a polyethylen glycol and a gelatin microparticles layer promisingly were found to differentiate into osteoblasts or chondrocytes in the respective layer, TGFβ1 supplementation of the gelatin layer and presence of osteoblastic cells in the polyethylen glycol layer both having a supportive effect on chondrogenesis [54]. Moreover, the inductive effect our hydrogels have on spontaneous adMSC spheroid formation are likely to promote chondrogenic differentiation, since rat bmMSC embedded in gelatin hydrogel microspheres releasing TGFβ1 were found to more strongly produce glycosaminoglycans than bmMSC on gelatin sheets [55]. Therefore, the gelatin-based hydrogels we produced are promising candidates for subsequent *in vivo* studies aiming at disease-state cartilage regeneration.

## Figures and Tables

**Scheme 1.**
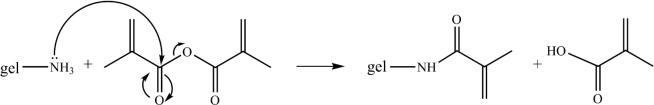
Modification of gelatin with methacrylic anhydride.

**Table 1.** Sequences of primers used in real-time RT PCR.

Gene	Accession Number	Forward Primer (5’-3’)	Reverse Primer (5’-3’)
*ACTB*	NM_001101.2	cttcctgggcatggagtc	agcactgtgttggcgtacag
*SOX5*	NM_178010.1	aagcctttcctgacatgcac	tccagcgagatcccaatatc
*SOX9*	NM_000346.2	tgggcaagctctggagac	cgttcttcaccgacttcctc
*COL1A1*	NM_000088.3	acgaagacatcccaccaatc	agatcacgtcatcgcacaac
*COL2A1*	NM_033150.2	aatggtggcttccatttcag	gtgatgttctgggagccttc
*VEGFA*	NM_001025366.1	ccttgctgctctacctccac	aatctgcatggtgatgttgg

**Table 2.** Atomic surface composition (in atomic %) of gelatin and methacrylamide-modified gelatin (gel-MOD) obtained by means of X-ray photoelectron spectroscopy.

Element	Gelatin (%)	Gel-MOD (%)
**C**	69	72
**N**	13	10
**O**	18	18
**N/C**	0.19	0.12
**N/O**	0.72	0.56
